# Household Food Insecurity, Dietary Diversity, Stunting, and Anaemia among Left-Behind Children in Poor Rural Areas of China

**DOI:** 10.3390/ijerph16234778

**Published:** 2019-11-28

**Authors:** Qiping Yang, Tong Yuan, Lina Yang, Jiaojiao Zou, Meimei Ji, Yefu Zhang, Jing Deng, Qian Lin

**Affiliations:** 1 Department of Nutrition Science and Food Hygiene, Xiangya School of Public Health, Central South University, Changsha 410078, China; yangqiping12@csu.edu.cn (Q.Y.); yuantong@csu.edu.cn (T.Y.); ylnly1997@csu.edu.cn (L.Y.); zjj227@csu.edu.cn (J.Z.); jimeimei@csu.edu.cn (M.J.); zhangyefu@csu.edu.com (Y.Z.); 2 Department of Epidemiology and Statistical Science, Xiangya School of Public Health, Central South University, Changsha 410078, China

**Keywords:** food insecurity, DDS, stunting, anaemia, left-behind children, China

## Abstract

Left-behind children (LBC) are a newly emerged social group in China. Poor nutritional status is particularly prominent in this population. However, their food insecurity tends to attract very little attention. This study aims to investigate the relationship between food insecurity and undernutrition (stunting and anaemia) in 3 to 5-year-old LBC in rural China. Face-to-face interviews were administered to 553 LBC caregivers in 40 rural villages of Hunan Province, China. The Household Food Insecurity Access Scale (HFIAS) was used to assess household food insecurity (HFI). Dietary diversity score (DDS) and food group consumption frequency were measured by 24 h-recall and food frequency questionnaires (FFQ). Hemoglobin tests and anthropometric measurements including height and weight were measured by trained health professionals. Logistic regression was constructed to assess the association between household food insecurity and dietary diversity, stunting, and anaemia. A high prevalence of household food insecurity was determined (67.6%). The weighted prevalence of stunting and anaemia were 16.6% and 26.5%, respectively. Food insecurity was positively associate with LBC stunting (severe HFI: OR = 6.50, 95% CI: 2.81, 15.00; moderate HFI: OR = 3.47, 95% CI: 1.60, 7.54), and anaemia (severe HFI: OR = 1.91, 95% CI: 1.02, 3.57). LBC with food insecurity had significantly lower dietary diversity than those who were food-secure (*p* < 0.001). The prevalence of household food insecurity among LBC in poor rural China is high and is associated with low DDS, stunting, and anaemia. Nutritional intervention programs and policies are urgently needed to reduce household food insecurity and undernutrition for this vulnerable population.

## 1. Introduction

Child undernutrition is a major worldwide health concern, which often causes irreversible damage to the physical and mental health of children as well as adversely affecting health and productivity throughout adulthood. Due to lower socio-economic status and inadequate health facilities, rural areas of China may be facing a severe challenge of child stunting and anaemia, especially for left-behind children (LBC). The number of LBC in China has increased dramatically from 15.8 to 23.4 million between 2005 and 2013 because of the continually increasing population of migrant workers [1,2]. It is estimated that there are more than 61 million LBC in China based on national population census data in 2014, accounting for 37.7% of all rural children [3]. Living separately from parents, LBC are entrusted to their elderly grandparents or maternal grandparents, distant relatives, and even neighbors who have poor education, vulnerable economic status, and inability to provide adequate care for the LBC. As a result, LBC are weaker than the non-left-behind children in learning achievement, nutrition status, accessibility of basic health service, and physical and mental development [4,5,6,7,8]. National survey data from 2009 showed that in poverty-stricken areas, the rate of stunting and underweight in children under five years of age was 12.1%, while the rate of stunting in LBC was 20%–30% higher than the others [9]. A study in 2011 showed that the prevalence of iron deficiency anemia in LBC under five years in Hunan province was 34.3% [10], while the national survey in 2013 reported 11.6% among children under five years old in 30 provinces [11]. 

Food insecurity has been identified as a key underlying cause of child malnutrition [12] and defined as “a household-level economic and social condition of limited or uncertain access to adequate food” [13]. The household food insecurity access scale (HFIAS) has been widely used to estimate the prevalence of household food insecurity (HFI), which is still a major public health problem worldwide [14]. HFI may lead to inadequate dietary intake, limited choice of food, and affect children’s nutritional status by compromising their dietary intake quantity and diet quality. Some studies showed that dietary diversity is an important index to reflect diet quality and an independent factor positively associated with child nutritional status [15,16]. However, mixed results were found in other empirical studies [17,18]. Socioeconomic factors, access to health service, water sanitation, and parental care are also important factors related to child nutritional status. Therefore, further studies are needed to explore the potential pathway of food insecurity and child undernutrition in different cultural backgrounds. 

The association between household food insecurity and undernutrition has been confirmed in a number of studies [19,20,21], but the food insecurity in rural Chinese LBC has not been evaluated. There are presently only two published studies focused on household food insecurity in China. One involved primary school students in poor rural areas [22] and the other, the elderly in Beijing [23], with the prevalence of household food insecurity being 6.1% and 54.2%, respectively. Given this gap in the evidence, the aim of this study is to assess household food insecurity among 3 to 5-year-old LBC in poor rural areas of China to explore the association between household food insecurity and LBC’s dietary diversity, and LBC undernutrition (stunting and anemia). We hypothesize that HFI is high in rural LBC households and HFI is positively associated with low dietary diversity, stunting, and anaemia. 

## 2. Method

### 2.1. Ethical Approval

This research was approved by the independent ethics committee of the Institute of Clinical Pharmacology, Central South University (ctxy-140003), and was registered in the China Clinical Trial Register (ChiCTR-TRC-14005117). Eligible subjects were identified with the assistance of a local village doctor. Caregivers were informed of the study and instructed to go to the village clinic at an appointed time. Written informed consent was obtained from all participants.

### 2.2. Participant Recruitment and Enrollment

The present study data were drawn from the baseline study of the project “Health allowance for improving nutritional status and development of the 3–5 y left-behind children in poor rural areas of China: a cluster randomized trial”. The baseline study was carried out among a sample of 610 rural children aged 3 to 5 years, from two geographical areas of rural Hunan, China in 2015. The selection process of setting and participants was described in our previous article [24]. In this study, 57 children were excluded for not having complete data on food insecurity or anthropometric measurements or valid dietary intake assessments, and 553 participants remained in the current analysis.

### 2.3. Outcome Variables 

The primary outcome variables were stunting and anaemia. The height of children was measured by the TB14-65-type height meter without shoes and hats. Height measurement data was calculated by WHO Anthro software to assess the height-for-age Z score (HAZ) [25]. Stunting is defined by height-for-age Z score (HAZ) <–2 SD of the WHO Child Growth Standards median. Hemoglobin tests were used to identify anaemia measured by trained health professionals. The diagnosis of anaemia in children 6–59 months of age is based on a hemoglobin of less than 11.0 mg/dL [26].

### 2.4. Assessment of Household Food Insecurity

Household food insecurity (HFI) was assessed by the Household Food Insecurity Access Scale (HFIAS), which was developed by the United States Agency for International Development. HFIAS is a simple, easy-to-use, highly applicable tool to measure household food insecurity, which reflect the food security situation of children in the family [27]. The scale includes nine occurrence questions and nine frequency-of-occurrence questions, with scores ranging from 0 to 27 points. As Table 1 shows, household food insecurity can be categorized into four degrees: food security, mild food insecurity, moderate food insecurity, and severe food insecurity. HFIAS has shown acceptable validity and applicability in different cultural backgrounds [28,29,30] but has not been validated in Chinese studies [22,23].

### 2.5. Assessment of Dietary Intake

Dietary diversity score (DDS) and consumption of food groups were assessed by 24 h-recall and food frequency questionnaires (FFQ) by trained investigators with a nutrition background. A 24 h-recall was used to collect information on all food the LBC consumed in the previous 24 h. Two questions “Did your child eat like usual?”, “A lot more or a lot less than usual?” were asked when a 24-hour recall was completed. The data were excluded if the LBC ate a lot more or a lot less than usual. According to the Chinese Balanced Diet Pagoda, the foods consumed were then aggregated into nine food groups: grains, cereals, and other starchy staples; dark green leafy vegetables; other vegetables; fruits; meat and poultry; fish and shrimp; dairy products; eggs; legumes and nuts. One point was taken into count for any food group consumed above 15 grams and at least once per day, and the DDS range from 0–8 points. A higher DDS reflects a more diverse diet [31,32]. In this study, a cutoff point of < 5 (median of DDS) was defined as low DDS of LBC.

The consumption frequency of each food group in the previous week was also assessed by a food frequency questionnaire (FFQ) with 18 food categories, which is based on an FFQ25 which has been previously validated [33]. There are ten frequency alternatives in total and the intervals range from “never” to “three times a day or more”.

### 2.6. Socioeconomic and Demographic Characteristics

A face-to-face interview was used to collect the socioeconomic and demographic characteristics of the study population through, including region, ethnicity family size, the number of LBC in the family, general information of the LBC (age, sex and left-behind status), general information of main caregivers (age, sex, education level, career, relationship to the LBC), socioeconomic status (e.g., annual household income, type of house, water source). 

### 2.7. Statistical Analysis

EpiData 3.0 software (The EpiData Association, Odense, Denmark) was used for data entering and sorting. Continuous and categorical variables in different groups were compared by ANOVA and chi-square test, respectively. A binary logistic regression was used to assess household food insecurity in relation to the LBC stunting in multivariate models, adjusted for region, economic level, the LBC’s sex, ethnicity, left-behind status, number of LBC, caregiver’s age, caregiver’s education level, caregiver’s relationship to LBC, and DDS. Data were analyzed by using IBM SPSS 18.0 software package (IBM Corp., Armonk, NY, USA). *p*-Values ≤ 0.05 at 95% confidence interval were considered statistically significant. 

## 3. Results

### 3.1. General Characteristics and Household Food Insecurity

The characteristics of the LBC included in this study are presented in Table 2. A total of 553 LBC were obtained complete data, including 307 boys (55.5%) and 246 girls (44.5%) aged 4.14 ± 0.90 years. Most of the LBC observed were of Han ethnicity (61.8%) and had both parents working outside the home (74.7%). We found that the prevalence of HFI was 67.6% among the observed LBC, while 15.4%, 33.4%, and 18.8% of them had mild, moderate, and severe household food insecurity, respectively. This study also found that 63.5% of the LBC did not fulfill the cut-off of low DDS (cut-off = 5). In moderate and severe HFI families, nearly 70% and 85% of the LBC obtained ≤ 4 DDS, respectively.

Of the CLBC, 83.9% were the LBC’s grandparents, 67.3% were female, 89.0% were farmers, and 30.2% never had any formal education. Most of the caregivers (73.2%) took care of two or more LBC. A chi-square test showed there were significant differences in household food insecurity of the LBC at regional, household SES, ethnicity, LBC’s DDS, and CLBC’s education levels (all *p* < 0.01).

### 3.2. Household Food Insecure Status and LBC’s Dietary Diversity, Dietary Consumption Frequency 

**Figure 1:**
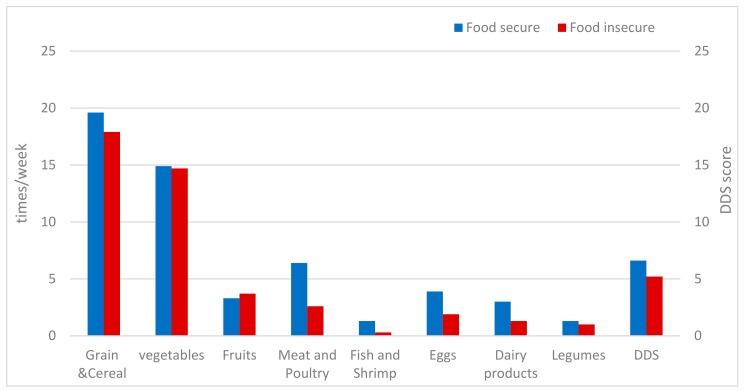
Dietary diversity score (DDS) and weekly food consumption frequencies by household food security status.

Figure 1 demonstrates the weekly consumption frequency of food groups and the DDS of the observed LBC. Among rural LBC, the most commonly consumed food groups were grain, cereals, and vegetables. LBC in food-secure households had a higher consumption frequency of animal source food than the food-insecure (meat and poultry (6.4 vs. 2.6), fish and shrimp (1.3 vs. 0.3), eggs (3.9 vs. 1.9), and dairy products (3 vs. 1.3)). LBC in food-insecure households consumed limited types of foods and had a lower DDS score than the food-secure (6.61 vs. 5.2). 

Compared with the food-secure, the odds of low DDS were significantly higher among LBC with severe, moderate and mild HFI, with an AOR of 17.129 (95% CI: 7.414, 39.572), 3.585 (95% CI: 1.961, 6.554), and 2.321 (95% CI: 1.143, 4.714), respectively. Those who are minorities were 2.1 times more likely to have low dietary diversity (AOR = 2.143, 95% CI: 1.260, 3.644). Low socioeconomic status is associated with low DDS with an AOR of 2.063 (95% CI: 1.121, 3.799). LBC whose caregivers were female or with higher education were less likely to have low dietary diversity, with an AOR of 0.048 (95% CI: 0.027, 0.086), and 0.274 (95% CI: 0.134, 0.562), respectively (Table 3).

### 3.3. Stunting and Anaemia by Household Food Insecure Status and DDS

**Figure 2:**
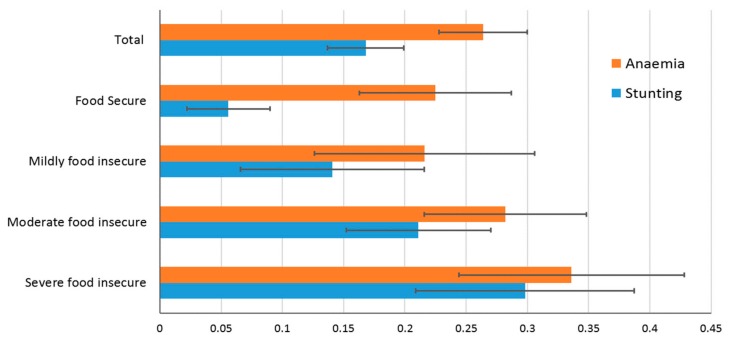
Distribution of prevalence and 95% confidence intervals for stunting and anaemia among left-behind children by household food insecurity status.

Figure 2 shows the prevalence of stunting and anaemia of observed LBC with different HFI status. For the total study population, the weighted prevalence rates for stunting, wasting, and underweight were 12.8%, 1.3%, and 2.0%, respectively. Stunting and anaemia were more prevalent in LBC in severe or moderate food-insecure households (29.8% or 21.1% for stunting and 33.6% or 28.2% for anaemia) than those LBC who lived in food-secure households (5.6% for stunting and 22.6% for anaemia). 

**Figure 3:**
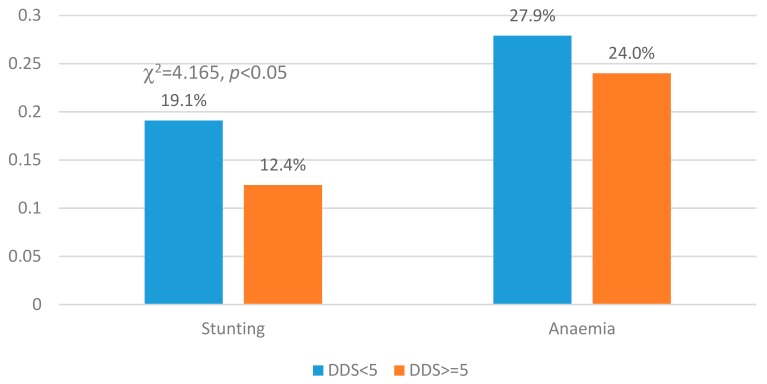
Stunting and anaemia by different DDS levels, differences were analyzed using chi-squared tests.

Figure 3 shows the stunting and anaemia of LBC at different DDS levels. Stunting was more prevalent in LBC with lower DDS than those with higher DDS (19.1% vs. 12.4%, χ^2^ = 4.165, *p* < 0.05). No significant difference was observed for anaemia. 

### 3.4. Association between LBC Stunting and Anaemia and Household Food-Insecure Status

Table 4 shows the unadjusted and adjusted ORs for assessing the association between stunting, anaemia of LBC and their household food insecurity status. We adjusted for different variables in three logistic regression models (Model 1, adjusted for LBC’s age and gender; Model 2, adjusted for covariate factors in Model 1 plus ethnicity, region, left-behind status, and the LBC’s DDS; Model 3, adjusted for covariate factors in Model 2 plus caregivers’ education level, household economic level, caregivers’ age and caregivers’ relationship to the LBC). Results from multivariable-adjusted logistic regression model suggest that LBC from severely food-insecure households were 6.49 times more likely to be suffering from stunting (OR = 6.49, 95% CI: 2.81, 15.00), and those from moderately food-insecure household were 3.47 times more likely to be stunted (OR = 3.47, 95% CI: 1.60, 7.54) compared those LBC who live in food-secure households. We also found that LBC with severe household food insecurity were 1.91 times more likely to suffer from anaemia than LBC in food-secure households (OR = 1.91, 95% CI: 1.02, 3.57). 

## 4. Discussion

People living in poverty environments are more prone to limited food access, but food insecurity prevalence has disparities among regions. In the present study, the HFI in observed LBC (67.6%) was higher than many other populations. In 2018 [34], 11.1 percent of U.S. households were food-insecure (14.3 million households). Households with food insecurity among children in 2018 (7.1 percent) was lower than in any year, but there were still more than 11 million children. A study in 2011 among 1583 elementary school students aged 6–14 years reported the prevalence of food insecurity among Chinese rural primary students was 6.1%, and 16.3% in participants with severe malnutrition [22]. UNICEF reported that the global prevalence of food insecurity for households with children under age 15 was 41%, and HFI was the lowest in East Asia/Pacific [35]. It was reported that the prevalence of household food insecurity within European countries was reported to be from 10% to 22% [36,37]. Some studies in sub-Saharan African developing countries have shown that the prevalence of household food insecurity can reach over 80% [38]. Household food security may be an essential element of adequate dietary intake that leads to a good nutritional status. Our study shows that about 64% of LBC have low dietary diversity (DDS < 5), which is much lower than that of their peers [32,39]. Stunting is more prevalent in LBC with lower DDS in HFI families, which means the LBC in HFI families were more likely to be consuming inadequate diets than those in food-secure households. Compared with other countries or regions, the food insecurity of left-behind children in poor areas of China should receive more attention and support, and it can be improved.

The high prevalence of HFI and low DDS in our observed population can be explained by their demographic characteristics. The most important determinants of HFI is socio-economic status [20]. Data from previous studies have routinely shown household income to be the most consistent and strongest predictor of the risk of food insecurity [40,41]. We found the proportion of grandparents or maternal grandparents caring for LBC was up to 83.9%, and they were mostly economic dependents with limited financial resources. Households with lower economic levels were more vulnerable to HFI and may tend to reduce the purchase of high-priced but high-quality protein food, such as animal source foods. In this study population, the main caregivers’ education level and household income were negatively associated with low DDS and HFI, which is consistent with several previous studies [14,23]. The rural caregivers have poor nutritional knowledge and are less likely to make proper food choices because of the lack of education, which was reported in another article about this observed population [42]. Traditional dietary customs also should be considered as one determinant. In this study, minorities were more likely to have low dietary diversity than the Han population. We found most minorities kept their traditional custom of two meals a day and they did not eat eggs. Furthermore, this study was conducted in winter when the households were more likely to be food-insecure due to a large shortage of food availability in rural areas. Rural households usually buy food at the local town markets (4–5 times a month). Because of bad weather, the yields of home-grown production (such as vegetables and crops) might be reduced. 

After adjusting for DDS and other confound factors, the results show that there is a significant association between HFI and LBC undernutrition (stunting and anaemia). We found that LBC in severe HFI households were more likely to being stunted or anaemia (OR = 6.49 or 1.91) than LBC in food-secure households. LBC in HFI households are likely to suffer malnutrition because of very limited intake of nourishing food. The decline in their dietary diversity is primarily due to lower consumption of meat and poultry, fish and shrimp, eggs and dairy products. We also find that the frequency consumption of animal source food in HFI children is lower than those who are food-secure, similar to other research results [43,44]. Another study conducted in 2009 showed that the dietary structure of left-behind children was inadequate. Energy and a variety of nutrient intake were not enough [45]. The lack of accessibility to food caused by household food insecurity might be a possible cause of stunting in left-behind children.

Food insecurity does not exist in isolation, as low-income families are affected by multiple, overlapping issues such as the lack of affordable housing, social isolation, chronic or acute health problems, high medical costs, and low wages. These issues are important social determinants of health, defined as the “conditions in the environments in which people are born, live, learn, work, play, worship, and age that affect a wide range of health, functioning, and quality-of-life outcomes and risks.” Our research population is left-behind children aged 3–5 years in rural areas of China. In this population, they should have the good care of their parents and enough nutritious food to promote growth. However, the fact is that they face food insecurity often or sometimes. Food insecurity is especially harmful to health during early childhood [14] and gives rise to risky behaviors and mental health issues during the transition to their adolescence and adulthood [46]. These LBC who experience household food insecurity may have a big impact on their life trajectory. 

Public policy interventions have been shown to reduce food insecurity and reach large numbers in the population in high-income countries [47]. From 2011 to 2018, the US food insecurity rate fell from 14.9 to 11.1 percent. About 56 percent of food-insecure households in the survey reported that, in the previous month, they had participated in one or more of the three largest federal nutrition assistance programs (SNAP, the Special Supplemental Nutrition Program for Women, Infants, and Children (WIC); and the National School Lunch Program) [34]. In recent years, the stunting and anaemia among rural LBC decreased because of the benefits from the increase in regional economic and policy support and universal basic public health services. The Chinese government has also implemented some LBC-related programs, such as the Integrated Nutrition Package for Rural Infants (<3 years old), the Nutrition Education for Infant Feeding Practice, and the Giving Priority to LBC in the Nutrition Improvement Program for School-Age Children (≥6 years old) [11,12,13,14]. In contrast, research on community-level interventions, such as food banks and other food programmes, suggests limited impact [30,48]. Although we have seen certain achievements in China in health promotion, the prevalence of undernutrition of LBC remains high in poor rural areas in China. In this study, the prevalence of stunting and anemia were 16.6% and 26.5%, which is higher than those of other children groups [49,50,51]. Nevertheless, here it deserves the attention of policymakers and researchers that there are currently no food security improvement projects for the LBC living in poor areas. Education and training for caregivers may decrease food insecurity by increasing their awareness of nutrition and proper food choices. Quality childcare institutions are also important for improving food security for young LBC, but those are very limited in rural areas. These challenges call for policymakers, health care providers, and various government departments to collaborate on developing innovative initiatives programs that improve food security for rural LBC.

Several limitations should be noted when interpreting our results. First, our investigation employed a cross-sectional design in two rural areas in southern China. As a result, we are unable to report causality in our results, and our findings may not be representative of the entire country. Second, we used self-reported data from the main caregivers of the LBC, which are subject to social-desirability and other forms of response bias; for example, respondents in this survey are mainly elders with low-levels of education and are ethnic minorities. Therefore, the respondents’ understanding of the questionnaire items might have been impacted by the different education levels, comprehension ability, and cultural backgrounds. Finally, this study used the HFIAS tool to measure the food insecurity status; although studies conducted in some developing countries have shown the HFIAS is an effective assessment of household food insecurity [28,30], the scale has never been validated in China. From a Chinese cultural background, the reliability, validity, and sensitivity have not been verified, and more follow-up studies are needed. 

Despite these limitations, this study makes an important contribution to our understanding of the associations between food insecurity and child health in rural areas in the developing world. Our study points to household food insecurity as being important for a child’s health, net of socioeconomic and demographic conditions. While reducing household insecurity may require long-term strategies, improving access to nutrient-dense food in low education and socioeconomic status contexts may provide benefits to child health in the short term. Further, even smaller improvements in the household food environment, such as moving households from a mildly-insecure to a food-secure status, can benefit child health. To our knowledge, this is the first study to assess the prevalence of food insecurity in left-behind children in rural China. Further studies are needed to evaluate food insecurity and social determinants in this population.

## 5. Conclusions

In summary, there is a high prevalence of food insecurity among households with 3–5-year-old LBC in poor rural areas of China. Household food insecurity is associated with the LBC’s low DDS, stunting, and anaemia. Further studies are needed to identify HFI in this vulnerable population to develop evidence-based public health intervention programs or policies. 

## Figures and Tables

**Table 1:** Categories of household food insecurity.

Question	Frequency		
	Rarely: 1	Sometimes: 2	Often: 3
1a			
2a			
3a			
4a			
5a			
6a			
7a			
8a			
9a			

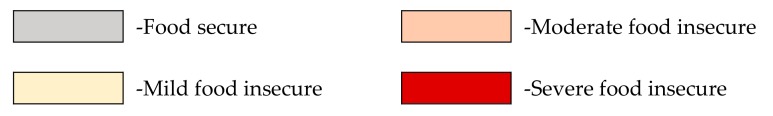

**Table 2:** Household food insecurity status by socio-demographic characteristics (n = 553).

Variables ^†^	Household Food Secure	Mild Food Insecure	Moderate Food Insecure	Severe Food Insecure	Total	*p*-Value ^#^
N, %	179, 32.4%	85, 15.4%	185, 33.4%	104, 18.8%	553, 100%	
Region (n, %)						
Mountain area	53, 29.6%	48, 56.5%	113, 61.1%	61, 58.7%	275, 49.7%	<0.01
Gentle hills	126, 70.4%	37, 43.5%	72, 38.9%	43, 41.3%	278, 50.3%	
Household SES level (n, %) (tertiles) *						
Low	35, 19.6%	25, 29.4%	71, 38.4%	55, 52.9%	186, 33.6%	<0.01
Middle	63, 35.2%	31, 36.5%	61, 33.0%	28, 26.9%	183, 33.1%	
High	81, 45.2%	29, 34.1%	53, 28.6%	21, 20.2%	184, 33.3%	
LBC’s age (year)	4.2 ± 0.9	4.1 ± 0.9	4.1 ± 0.9	4.1 ± 1.0	4.1 ± 0.9	0.920
LBC’s sex (n, %)						
Boy	107, 59.8%	42, 49.4%	104, 56.2%	54, 51.9%	307, 55.5%	0.365
Girl	72, 40.2%	43, 50.6%	81, 43.8%	50, 48.1%	246, 44.5%	
Ethnicity (n, %)						
Han	144, 80.4%	50, 58.8%	85, 45.9%	63, 60.6%	342, 61.8%	<0.01
Minorities	35, 19.6%	35, 41.2%	100, 54.1%	41, 39.4%	211, 38.2%	
Left-behind status (n, %)						
Single parent out	35, 19.6%	18, 21.2%	56, 30.3%	31, 29.8%	140, 25.3%	0.059
Both parents out	144, 80.4%	67, 78.8%	129, 69.7%	73, 70.2%	413, 74.7%	
LBC’s DDS						
0–4	79, 44.1	54, 63.5	130, 70.3	88, 84.6	351, 63.5	<0.01
≥5	100, 55.9	31, 36.5	55, 29.7	16, 15.4	202, 36.5	
Number of LBC in the family (n, %)						
1	54, 30.2%	21, 24.7%	48, 25.9%	25, 24.0%	148, 26.8%	0.205
2	84, 46.9%	31, 36.5%	86, 46.5%	51, 49.0%	252, 45.6%	
3 and above	41, 22.9%	33, 38.8%	51, 27.6%	28, 26.9%	153, 27.7%	
CLBC’s age (year)	54.6 ± 12.0	56.0 ± 11.6	56.0 ± 12.7	56.0 ± 12.0	55.6 ± 12.1	0.677
CLBC’s sex (n, %)						
Male	57, 31.8%	27, 31.8%	60, 32.4%	37, 35.6%	181, 32.7%	0.921
Female	122, 68.2%	58, 68.2%	125, 67.6%	67, 64.4%	372, 67.3%	
CLBC’s relationship to LBC (n, %)						
Mother	12, 6.7%	6, 7.1%	22, 11.9%	13, 12.5%	53, 9.6%	0.409
Father	8, 4.5%	4, 4.7%	8, 4.3%	4, 3.8%	24, 4.3%	
Grandparent	152, 84.9%	73, 85.9%	152, 82.2%	887, 83.7%	464, 83.9%	
Other	7, 3.9%	2, 2.4%	3, 1.6%	0, 0.0%	12, 2.2%	
CLBC’s education level (n, %)						
No formal education	31, 17.3%	31, 36.5%	69, 37.3%	36, 34.6%	167, 30.2%	< 0.01
Primary school	92, 51.4%	43, 50.6%	84, 45.4%	48, 46.2%	267, 48.3%	
Middle school and above	56, 31.3%	11, 12.9%	32, 17.3%	20, 19.2%	119, 21.5%	
CLBC’s career (n, %)						
Non-farmer	25, 14.0%	6, 7.1%	14, 7.6%	16, 15.4%	61, 11.0%	0.066
Farmer	154, 86.0%	79, 92.9%	171, 92.4%	88, 84.6%	492, 89.0%	

^†^ LBC, left-behind children; CLBC, caregiver of left-behind children; SES, socioeconomic status. ^#^ *p*-Values from ANOVA for continuous variables, chi-square test for nominal variables. * SES was estimated following principal component analysis, including various items related to the economic status: family size, household annual income, size of land used for cultivation, housing type, access to tap water, and number of bedridden patients at home.

**Table 3:** HFI and socioeconomic variables associated with low DDS of LBC (N = 553).

Variables	COR	(95% CI)	AOR	(95% CI)
HFI (reference: food-secure)				
Mildly food-insecure	2.245 *	(1.098–4.589)	2.321 *	(1.143–4.714)
Moderate food-insecure	3.511 ***	(1.917–6.428)	3.585 ***	(1.961–6.554)
Severely food-insecure	17.020 ***	(7.306–39.650)	17.129 ***	(7.414–39.572)
Minorities	2.084 **	(1.216–3.572)	2.143 **	(1.260–3.644)
Socioeconomic status (reference: High)				
Middle	1.591	(0.932–2.718)	1.582	(0.932–2.684)
Low	2.020 **	(1.093–3.735)	2.063 *	(1.121–3.799)
Caregiver’s sex (reference: male)	0.046 ***	(0.025–0.083)	0.048 ***	(0.027–0.086)
Caregiver’s education (reference: no formal education)				
Primary school	0.665	(0.365–1.210)	0.656	(0.363–1.185)
Middle school	0.277 **	(0.135–3.572)	0.274 ***	(0.134–0.562)

Notes: Logistic regression was applied in analysis; * *p* < 0.05, ** *p* < 0.01, *** *p* < 0.001; HFI: household food insecurity; COR: crude odds ratio; AOR: adjusted odds ratio, adjusted for LBC age, sex, region, caregiver’s age and caregiver’s relationship to LBC.

**Table 4:** Odds ratios and 95% confidence intervals for the association between food insecurity and LBC stunting, and anemia.

Variables	Household Food Insecurity Status OR (95%CI)			
	Food Secure	Mild Food Insecure	Moderate Food Insecure	Severe Food Insecure
Stunting				
Crude	1	2.778 (1.149–6.718) ***	4.514 (2.177–9.360) ***	7.177 (3.344–15.404) *
Model 1 ^†^	1	2.925 (1.205–7.099) ***	4.648 (2.236–9.660) ***	7.575 (3.511–16.343) *
Model 2 ^‡^	1	2.454 (0.987–6.100)	3.746 (1.743–8.049) **	6.798 (2.971–15.556) ***
Model 3 ^§^	1	2.251 (0.896–5.658)	3.468 (1.596–7.537) **	6.495 (2.812–15.002) ***
Anemia				
Crude	1	0.948 (0.505–1.780)	1.349 (0.838–2.173)	1.737 (1.014–2.975) *
Model 1 ^†^	1	0.974 (0.517–1.832)	1.370 (0.849–2.209)	1.787 (1.040–3.069) *
Model 2 ^‡^	1	1.074 (0.560–2.061)	1.540 (0.920–2.576)	1.903 (1.047–3.458) *
Model 3 ^§^	1	1.057 (0.538–2.076)	1.591 (0.932–2.714)	1.912 (1.025–3.566) *

Notes: Multivariable-adjusted logistic regression model was applied in analysis; * *p* < 0.05, ** *p* < 0.01, *** *p* < 0.001; ^†^ Model 1, adjusted for LBC age and gender; ^‡^ Model 2, adjusted for the LBC’s age, gender, ethnicity, region, left-behind status and the LBC’s DDS; ^§^ Model 3, adjusted for covariate factors in Model 2 plus caregivers’ education level, household economic level, caregivers’ age and caregivers’ relationship to the LBC.
